# 521. Investigating the Gram-Positive Selective Spectrum of Ibezapolstat, a First-in-Class DNA Polymerase IIIC (Pol IIIC) Inhibitor

**DOI:** 10.1093/ofid/ofac492.576

**Published:** 2022-12-15

**Authors:** M Jahangir Alam, Khurshida Begum, Md Ekramul Karim, Chenlin Hu, Eugenie Basseres, Chris Lancaster, Kevin W Garey

**Affiliations:** University of Houston College of Pharmacy, Houston, Texas; University of Houston College of Pharmacy, Houston, Texas; University of Houston College of Pharmacy, Houston, Texas; University of Houston, Houston, Texas; University of Houston College of Pharmacy, Houston, Texas; University of Houston, Houston, Texas; University of Houston College of Pharmacy, Houston, Texas

## Abstract

**Background:**

Ibezapolstat (IBZ) is a non-absorbable antimicrobial currently in phase 2 clinical trials for the treatment of *Clostridioides difficile* infection (CDI). *In vitro* and human studies have shown potent activity of IBZ against *C. difficile* but selective activity against other beneficial Gram-positive gut microbiota shown to reduce the risk of recurrent CDI. As the target DNA Pol IIIC enzyme is present in most Gram-positive species, the reasons for this selectivity are unclear. The purpose of this study was to assess the selectivity of IBZ against Gram-positive gut microbiota.

**Methods:**

Using stool samples and microbiome data from the phase 1 and 2 studies, changes in proportional abundance of gut microbiome species were analyzed over time in healthy volunteers or patients with CDI given IBZ. Using a separate collection of gut microbiota species, MIC determinations against a variety of Gram-positive gut species were assessed by broth microdilution.

**Results:**

Baseline gut microbiota from healthy volunteers were primarily Firmicutes, Bacteroidetes, or Actinobacteria. Actinobacteria increased in abundance after starting IBZ (primarily *Bifidobacteriales* or *Coriobacteriales*) and persisted for the entire dosing period. In comparison to the phase 1 study, the phase 2a CDI study baseline microbiota had a lower proportion of Actinobacteria and Firmicutes and increased Bacteroidetes. In CDI patients, Actinobacteria increased in abundance after starting IBZ (primarily *Coriobacteriales*) followed within 2-3 days by decreased abundance of Bacteroidetes, and an increased abundance of Lachnospiraceae and Ruminococcaceae. Using isolated gut microbiota species, IBZ was inactive (MIC >64 µg/mL) against representative Actinobacteria (Bifidobacteriaceae and Coriobacteriaceae) and certain Firmicutes (Lachnospiraceae and Lactobacillaceae) but highly active against strains of *C. difficile* (MIC<2 µg/mL).

**Conclusion:**

Microbiome changes with IBZ were dependent on underlying composition of the baseline microbiome but consistently demonstrated increased abundance of Actinobacteria after starting therapy. IBZ microbiome data coupled with *in vitro* MIC determinations demonstrated persistence or regrowth of healthy microbiota associated with beneficial physiologic effects.

**Disclosures:**

**Kevin W. Garey, PharmD, MS**, Acurx Pharmaceuticals: Grant/Research Support|Paratek Pharmaceuticals: Grant/Research Support|Seres Therapeutics: Grant/Research Support|Summit Pharmaceuticals: Grant/Research Support.

